# Autopsy Findings and Causality Relationship between Death and COVID-19 Vaccination: A Systematic Review

**DOI:** 10.3390/jcm10245876

**Published:** 2021-12-15

**Authors:** Francesco Sessa, Monica Salerno, Massimiliano Esposito, Nunzio Di Nunno, Paolo Zamboni, Cristoforo Pomara

**Affiliations:** 1 Department of Clinical and Experimental Medicine, University of Foggia, 71122 Foggia, Italy; francesco.sessa@unifg.it; 2 Department of Medical, Surgical and Advanced Technologies “G.F. Ingrassia”, University of Catania, 95121 Catania, Italy; monica.salerno@unict.it (M.S.); massimiliano.esposito91@gmail.com (M.E.); 3 Department of History, Society and Studies on Humanity, University of Salento, 73100 Lecce, Italy; nunzio.dinunno@unisalento.it; 4 Vascular Diseases Center, Hub Center for Venous and Lymphatic Diseases Regione Emilia-Romagna, Sant’Anna University Hospital of Ferrara, 44121 Ferrara, Italy; zambo@unife.it

**Keywords:** COVID-19 vaccination, fatal case, adverse events following immunization (AEFI), vaccine-induced immune thrombotic thrombocytopenia (VITT)

## Abstract

The current challenge worldwide is the administration of the severe acute respiratory syndrome coronavirus 2 (SARS-CoV-2) vaccine. Considering that the COVID-19 vaccination represents the best possibility to resolve this pandemic, this systematic review aims to clarify the major aspects of fatal adverse effects related to COVID-19 vaccines, with the goal of advancing our knowledge, supporting decisions, or suggesting changes in policies at local, regional, and global levels. Moreover, this review aims to provide key recommendations to improve awareness of vaccine safety. All studies published up to 2 December 2021 were searched using the following keywords: “COVID-19 Vaccine”, “SARS-CoV-2 Vaccine”, “COVID-19 Vaccination”, “SARS-CoV-2 Vaccination”, and “Autopsy” or “Post-mortem”. We included 17 papers published with fatal cases with post-mortem investigations. A total of 38 cases were analyzed: 22 cases were related to ChAdOx1 nCoV-19 administration, 10 cases to BNT162b2, 4 cases to mRNA-1273, and 2 cases to Ad26.COV2.S. Based on these data, autopsy is very useful to define the main characteristics of the so-called vaccine-induced immune thrombotic thrombocytopenia (VITT) after ChAdOx1 nCoV-19 vaccination: recurrent findings were intracranial hemorrhage and diffused microthrombi located in multiple areas. Moreover, it is fundamental to provide evidence about myocarditis related to the BNT162B2 vaccine. Finally, based on the discussed data, we suggest several key recommendations to improve awareness of vaccine safety.

## 1. Introduction

Severe acute respiratory syndrome coronavirus 2 (SARS-CoV-2) has been identified as the causative agent of coronavirus disease 2019 (COVID-19) [1]. The virus rapidly spread around the world, leading to one of the most severe pandemics in human history: on 2 December 2021, there were 263,565,559 cases worldwide, with more than 5,225,667 confirmed deaths, affecting 223 countries [2]. Vaccination is undoubtedly the most effective tool for preventing infectious diseases, representing one of the most important breakthroughs in the history of medical science. To date, more than 4.29 billion vaccine doses have been administered worldwide, reaching about 55.9% of the global population. About 74% of these vaccinations have been administered in high- and upper-middle-income countries, and only 0.8% in low-income countries. In this context, it is important to note that several high-income countries are starting to receive an additional dose, while in low-income countries the number of fully vaccinated people is alarmingly low [3].

The development of COVID-19 vaccines started in January 2020 with the identification of the genetic sequence of SARS-CoV-2. Subsequently, many vaccine candidates were tested for the development of safe and effective COVID-19 vaccines, exploring different technologies such as mRNA, subunit proteins, and virus-based vaccines such as inactivated, live-attenuated, and recombinant viral vaccines. A new COVID-19 vaccine that uses circular strands of DNA to prime the immune system has recently been approved [4]. COVID-19 vaccination has been found to reduce the risk of SARS-CoV-2 transmission as well as hospitalization and associated complications [5]. This is attributed to vaccine efficacy through its ability to induce both humoral and cell-mediated immune responses in vaccinated subjects [6]. It has recently been reported that vaccines averted over one thousand deaths in Israeli during the first 4-months of the vaccination campaign [7]. Moreover, almost all patients hospitalized with COVID-19 by the end of May in Polish hospitals were not vaccinated [8]. Similar data have been published worldwide.

Following the recommendation of the European Medicines Agency (EMA), the European Union authorized the use of four vaccines, opening the way to a gradual return to pre-pandemic life. In particular, the vaccine BNT162b2 (Pfizer–BioNTech) was authorized on 21 December 2020 [9]; another vaccine, mRNA-1273 (Moderna), was approved on 6 January 2021 [10]; the third vaccine, ChAdOx1 nCov-19 (AstraZeneca), was approved on 29 January 2021 [11]; the fourth vaccine is Ad26.COV2.S (COVID-19 Vaccine Janssen—Johnson & Johnson), authorized on 11 March 2021 [12]. At the moment of writing, only these vaccines have been authorized for use in the European Union, while Sputnik V (Gam-COVID-Vac), COVID-19 Vaccine (Vero Cell), inactivated, and Vidprevtyn are currently under rolling review; finally, Nuvaxovid (also known as NVX-CoV2373) started the process of marketing authorization on 16 November 2021 [13].

It is important to note that the evaluation of adverse events following vaccination is a pivotal part of the clinical trials conducted pre-authorization. Moreover, clinical trials are not designed to detect very rare adverse events; this requires post-authorization monitoring. For example, in Europe, as reported on the EMA website, clinical trials are usually conducted on carefully selected patients and followed up very closely under controlled conditions. This means that at the time a medicine is authorized, as well as a vaccine, it has been tested on a relatively small number of selected patients for a limited length of time. For these reasons, it is essential that all medicines are monitored for safety throughout their use in healthcare practice. In this way, a pharmacovigilance system is mandatory after drug approval, monitoring suspected adverse reactions [14].

However, from a public health viewpoint, several important issues are still present and relevant in COVID-19 vaccines. They regard not only vaccine efficacy and protection duration, but also safety. In various countries, severe and fatal adverse effects occurring at the same time as COVID-19 vaccination have been reported [15], generating hesitancy and suspicion in the population [16,17].

Considering that COVID-19 vaccination represents the best possibility to resolve this the pandemic, this systematic review aims to clarify the major aspects of fatal adverse effects related to COVID-19 vaccines, with a further goal of advancing our knowledge supporting decisions or suggesting changes in policies at local, regional, and global levels. Moreover, this review aims to provide key recommendations to improve awareness of vaccine safety.

## 2. Materials and Methods

### 2.1. Search Strategy

The Preferred Reporting Items for Systematic Reviews and Meta-Analyses Statement (PRISMA) recommendations were applied to perform this systematic review [18]. We searched all publications related to SARS-CoV-2 vaccines and fatal adverse effects from the following databases: Scopus, EMBASE, Medline (via PubMed), and Web of Science. All studies published up to 2 December 2021 were searched without language restriction by three independent reviewers. Searched medical subject headings (MeSH) were: “COVID-19 Vaccine”, “SARS-CoV-2 Vaccine”, “COVID-19 Vaccination”, “SARS-CoV-2 Vaccination”, and “Autopsy” or “Post-mortem”. References and citation lists of selected articles and reviews were also reviewed for any other relevant literature.

### 2.2. Study Selection

The retrieved studies were first reviewed by three independent authors based on the title and abstract (FS, MS, and ME), all unrelated publications were removed, and the full texts of the remaining articles were fully reviewed. Then, two independent reviewers (CP and PZ) judged potentially eligible articles, and disagreements were resolved by discussion and for each article a consensus was reached.

### 2.3. Eligibility, Inclusion, and Exclusion Criteria

The following predetermined conditions had to be met for studies to be considered for inclusion in this meta-analysis. For initial screening, all studies with post-mortem investigations were included in the systematic review; English language was an inclusion criterion. All papers without post-mortem investigations, review articles, and studies with no extractable data were excluded from this review.

## 3. Results

**Figure 1:**
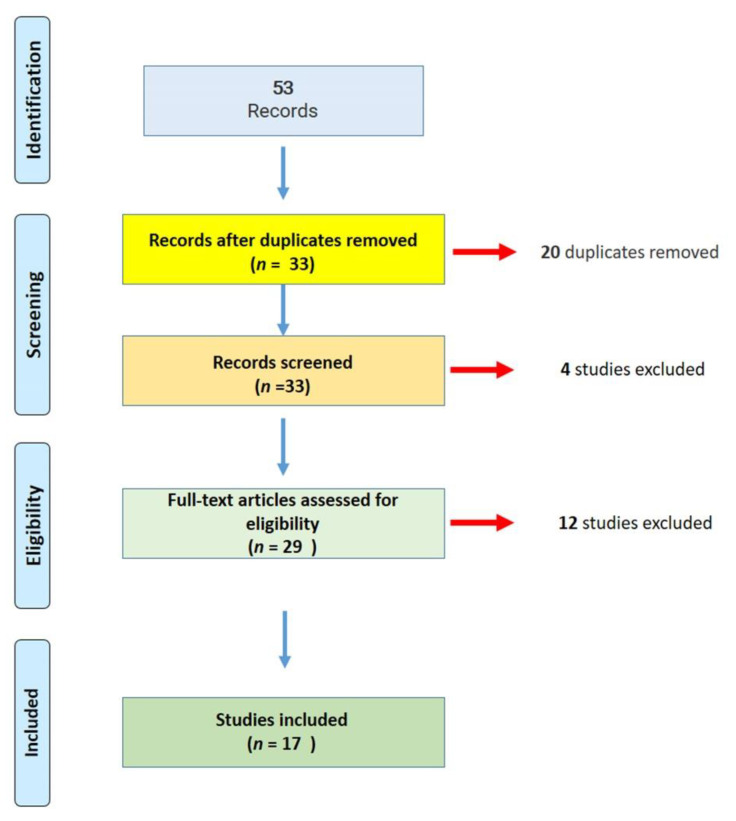
Flow diagram of the literature search and study selection for this systematic review (PRISMA flow chart).

A total of 53 publications matched the research parameters; removing duplicates, 33 articles were fully screened for COVID-19 vaccines and fatal adverse events. Out of these studies, 17 met the systematic review inclusion criteria (Figure 1).

As summarized in Table 1, 17 papers were published with fatal cases occurring at the same time as COVID-19 vaccine administration. A total of 38 cases (19 females, 19 males) were described: 22 cases were related to ChAdOx1 nCoV-19 administration, 10 cases to BNT162b2, 4 cases to mRNA-1273, and 2 cases to Ad26.COV2.S (Janssen).

**Figure 2:**
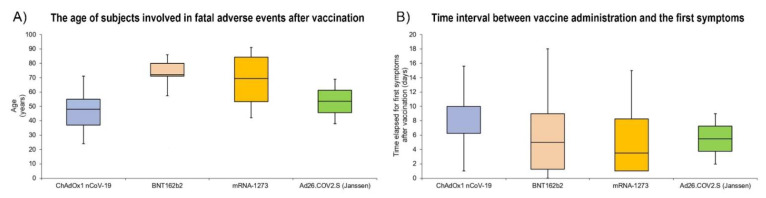
Box plot analysis comparing the age of subjects involved in fatal cases after vaccine administration (**A**). Comparison of the time interval between vaccine administration and the first symptoms (**B**).

Through a box plot analysis, we have summarized the data about the age of subjects involved in fatal adverse events after vaccination (Figure 2A), and the data about the time interval between vaccine administration and the first symptoms (Figure 2B).

Twenty-two (14 females, 8 males) cases were reported as deaths occurring at the same time as ChAdOx1 nCoV-19 administration. The mean age was 47.6 ± 12.28 (Male = 53.6 ± 13.9; Female = 44 ± 9.9).

Greinacher et al. [19] reported six cases of fatal adverse effects after COVID-19 administration, even if only one case is discussed in their report. Limiting the comments to this case, the authors reported a portal-vein thrombosis; moreover, they described thrombi in the splenic and upper mesenteric veins; finally, small thrombi were reported in the infrarenal aorta and both iliac arteries. Finally, autopsy findings revealed cerebral venous thrombosis. This paper described, for the first time, the presence of antibodies against platelet factor 4 (PF4), suggesting a similar pathological mechanism to severe heparin-induced thrombocytopenia (HIT).

Althaus et al. [20] discussed eight cases of death after ChAdOx1 nCoV-19 administration, although the post-mortem examination was performed in only two cases, as reported in Table 1. The main findings were massive cerebral hemorrhage with edema, and bilateral pulmonary thromboembolism. In both cases, the authors reported the presence of microthrombi on glomeruli. The authors concluded that all patients developed vaccine-induced immune thrombotic thrombocytopenia (VITT) after the administration of SARS-CoV-2 vaccine ChAdOx1 nCoV-19. This diagnosis was based on the presence of a high antibody titer against PF4. In this paper, the authors suggested that the presence of PF4 antibodies in VITT patients induced a significant increase in procoagulant markers.

Mauriello et al. [21] presented a fatal case of thromboembolism following administration of the first dose of ChAdOx1 nCOV-19 (AstraZeneca). At autopsy, massive cerebral hemorrhage was found, even if the level of serum anti-PF4 antibodies was undetectable. Based on their report, the authors suggested avoiding the use of ChAdOx1 nCOV-19 vaccine in subjects with a pre-existing condition of thrombocytopenia due to myelodysplasia, such as in the reported case.

Wieldmann et al. [22] presented a case series of five women with rapid progressive neurological symptoms, cerebral venous thrombosis (CVT) with intracerebral hemorrhage and thrombocytopenia, occurring 7/10 days after ChAdOx1 nCOV-19 (AstraZeneca) vaccination. Four of them died and autopsies were performed. The post-mortem findings are very similar in all subjects involved: cerebral hemorrhage with the presence of thrombi at the level of the sinuses. In all cases, the authors reported the presence of anti-PF4 antibodies.

Bjørnstad-Tuveng et al. [23] discussed a single case of a female healthcare worker who died of intracranial hemorrhage. Moreover, the authors described the presence of small thrombi in the transverse sinus, frontal lobe, and pulmonary artery. In light of the previous studies, the authors performed the anti-PF4 tests confirming the presence of these antibodies.

Scully et al. [24] reported seven cases, even if the post-mortem examination was performed in only one case, describing evidence of thrombosis in many small vessels located in the lungs, intestine, cerebral veins, and venous sinuses. Moreover, an extensive intracerebral hemorrhage and positivity for the anti-PF4 test were reported.

Günther et al. [25] described the case of a subject who presented with typical symptoms of VITT, including thrombocytopenia, cerebral venous and sinus thrombosis (CVST), and signs of disseminated intravascular coagulation (DIC). The presence of anti-PF4 antibodies was reported. The post-mortem findings confirmed the presence of residual thrombus in the left sinus transversus without evidence in the brain or in other organs.

Pomara et al. [26] presented two cases (one male and one female) of death after vaccine administration: the presence of extensive cerebral hemorrhages was reported in both cases. Moreover, in one case, portal and mesenteric thromboses with extension into the splenic vein were described, while, in the other case, massive thrombosis of the whole venous tree of the left upper limb extending from the hand to the axillary vein, with symmetric lesions in the veins of the right hand and the right axillary vein, was reported. In both cases, the anti-PF4 test was positive. It is important to note that for the first time the causality WHO algorithm was adopted to determine the direct link between vaccination and a fatal adverse effect [27]. Moreover, the same group suggested inserting autopsy as an essential tool that should be carried out in each suspected case.

Schneider et al. [28] discussed nine cases occurring at the same time as ChAdOx1 nCOV-19 vaccination: although they did not describe the application of the WHO algorithm to ascertain the causality relationship, the authors excluded it in one case, while they classified another case as “unlikely”, and the other two cases as “very likely”.

The fatal cases related to the BNT162b2 vaccine administration involved 10 subjects (7 females, 3 males), with an average age of 66.7 ± 20.8.

Edler et al. [29] described three cases of elderly subjects affected by severe cardiovascular diseases and other comorbidities (see Table 1). All subjects died in the context of these pre-existing conditions, while one case, testing positive at nasopharyngeal swab, developed COVID-19 pneumonia. In this report, it is important to note the pivotal role of autopsy in order to exclude a causality relationship between vaccine administration and death.

Hansel et al. [30] reported a case of an elderly male subject who had received the first dose of the BNT162b2 mRNA COVID-19 vaccine. The man was affected by several comorbidities, and although he did not present with any COVID-19-specific symptoms, he tested positive for SARS-CoV-2 before he died. The authors did not confirm the causality relationship.

Schneider et al. [28] discussed the data of five cases occurring at the same time as BNT162b2 mRNA COVID-19 vaccine administration. Based on pre-existing diseases and post-mortem findings they did not indicate a causal relationship with the vaccination. Only one case was classified as having a “possible” relationship with the vaccine administration.

Choi et al. [31] described a particular myocarditis related to the BNT162b2 mRNA COVID-19 vaccine, identifying histological differences from viral or immune-mediated myocarditis: indeed, the authors reported that the inflammatory infiltrates were predominantly neutrophils and histiocytes, rather than lymphocytes.

The fatal cases related to mRNA-1273 vaccine administration involved four subjects (two females, two males), with an average age of 68 ± 22.5.

Verma et al. [32] reported the first fatal case after mRNA-1273 vaccination: this is the first case related to the second rather than the first dose.

Schneider et al. [28] described three cases: the authors concluded that there was no relationship between death and vaccine administration based on the autopsy findings combined with pre-existing diseases.

The same authors reported one case related to the Ad26.COV2.S (Janssen) vaccine, reporting a possible causality relationship based on post-mortem findings. Similarly, Choi et al. [33] reported the fatal case of a subject who died two days after Ad26.COV2.S (Janssen) vaccination. Although the patient suffered from multiple myeloma diagnosed 1.5 years before, the cause of death was identified as fatal systemic capillary leak syndrome possible related to COVID-19 vaccination.

## 4. Discussion

Vaccination plays a key role in the pandemic war, representing a crucial measure of infection control [34,35]. At the time of writing, COVID-19 cases are sweeping Europe once again, particularly in those countries with a low rate of vaccination.

The first requirement is to ensure thorough, up-to-date, correct, and complete information on vaccines. In particular, their side effects must be publicized, including all useful information needed to interpret this properly in context [35]. Of course, in the case of the COVID-19 vaccination, the necessity of a promptly available vaccine has led to some adverse effects not being completely known. Although the rate of severe adverse effects is very low, it is important to highlight that in the first phase of vaccination, the package leaflet of each vaccine and the relative informed consent did not contain the unknown adverse effects that were added only after the first cases of severe adverse effects. It is important to remark that a pharmacovigilance system is mandatory after each drug approval, monitoring all suspected adverse reactions [14].

Based on the discussed data, a causality relationship between vaccine administration and death was demonstrated in 13 cases of ChAdOx1 nCOV-19 (AstraZeneca) vaccination, while it was excluded in the other 6 cases; in two cases the relationship was classified as “very likely”, and in the last one as “unlikely”. As concerns BNT162B2, of the ten cases reported in the literature, the causality relationship was established in one case, while in another case it was defined as “possible”. Finally, the causality relationship was established in one case of mRNA-1273 vaccination and classified as “possible” in the two cases related to the Ad26.COV2.S (Janssen) vaccine. As recently noted in a review published by Sharifian-Dorche et al. [36], other severe adverse effects have been described related to other authorized vaccines.

Analyzing the international data, it has been reported that both vaccines based on the adenoviral-based vector (ChAdOx1 nCov-19 and Ad26.COV2.S Janssen) can cause similar adverse reactions, generating severe adverse effects such as thrombocytopenia and thrombosis in atypical locations (cerebral and/or splanchnic veins) in healthy subjects a few days following vaccination. Based on the data obtained through this literature review, these symptoms appeared 8.6 ± 4.1 days after vaccine administration. All included cases were related to the first dose administration. Nevertheless, these severe adverse effects are extremely rare: 3 to 10 cases per million. Similar complications are lower for the two messenger RNA (mRNA)-based vaccines (BNT162b2 and mRNA-1273): severe adverse effects have been estimated to occur in 0.8 to 1 case per million [37].

The disclosure of any risks involved in vaccination is an integral part of the information provided: consent may only be effectively „informed” when the risks and benefits are completely understood. In the case of ChAdOx1 nCoV-19 we certainly cannot affirm that at the time of the first administrations the possible effects, such as those found (cerebral hemorrhages and diffuse thrombosis), were fully known. In this scenario, the first administration was completed in the absence of complete information for the patient: it is possible to make a risk assessment only when all adverse effects are known, and the risks are quantified based on research findings [38,39].

It is interesting to note that the criteria for the diagnosis of vaccine-induced death have been adopted only by Pomara et al. [26,27]: the authors adopted the proposed WHO algorithm to establish direct causality, confirming a direct link between vaccine administration and fatal adverse effects. As recently remarked by Mungmunpuntipamtip and Wiwanitkit [40], the criteria to establish a direct link between vaccination and fatal adverse effects should be standardized by the international community; in this way, the post-mortem investigation represents an essential tool to confirm all the data obtained during hospitalization.

The post-mortem investigation remains the gold standard to define the exact cause of death and the related pathophysiological processes [41,42]. The COVID-19 vaccine campaign began in about December 2020, and, at the same time, monitoring of death associated with adverse effects started in all countries. Although different fatal events have been reported occurring at the same time as COVID-19 vaccine administration, only a few papers have been published describing the post-mortem findings (38 cases: 22 patients were vaccinated with ChAdOx1 nCoV-19, 10 cases with BNT162b2, 4 cases with mRNA-1273, and 2 cases with Ad26.COV2.S Janssen), as summarized in Table 1. Based on these data, autopsy is very useful to define the main characteristics of the so-called VITT after ChAdOx1 nCoV-19 vaccination: the recurrent findings were intracranial hemorrhage and diffused microthrombi located in multiple areas. In two cases [19,26] brain hemorrhage was preceded by portal and mesenteric thrombosis with extension into the splenic vein. Microscopic evaluation was reported only in one study [27], showing several vascular thrombi and hemorrhagic areas at the level of the brain. In addition, diffuse thrombi were observed in small and medium-sized vessels due to endothelial activation after an inflammatory reaction with a procoagulant process and subsequent thrombotic reaction. The same group conducted immunohistochemical investigations, revealing the expression of adhesion molecules and activated inflammatory cells in the vascular and perivascular tissues of different organs (such as heart, lung, liver, kidney, ileum, and deep veins). The inflammatory cells were found to be arranged in clusters with aggregated platelets at the endoluminal level, confirming a pro-thrombotic state.

In addition, as described by Rzymski et al. [43], different mechanisms could be related to the severe/fatal adverse effects after ChAdOx1 nCoV-19 vaccination: the possible role of antibodies against platelet factor 4 (PF4); the direct interaction between adenoviral vector and platelets; the possible cross-reactivity of antibodies against SARS-CoV-2 spike protein with PF4; the possible cross-reactivity of anti-adenovirus antibodies and PF4; the possible interaction between spike protein and platelets; the platelet expression of spike protein and subsequent immune response; the platelet expression of other adenoviral proteins and subsequent immuno-reactions. Finally, considering the small number of subjects involved in similar adverse events, it is also plausible that thrombotic thrombocytopenia after COVID-19 vaccine administration may be multifactorial, with a pivotal role played by the genotype and/or influenced by post-transcriptional events. An important piece of data that emerges from this review is related to the age of the subjects involved in the fatal cases occurring at the same time as COVID-19 vaccination: while for the other vaccines the subjects involved were over 65 y.o., in the case of ChAdOx1 nCoV-19 the average age was 47.6, suggesting that the severe adverse effects occur more frequently in subjects under 65. This finding was made analyzing the average age in female subjects involved in fatal adverse effects related to the ChAdOx1 nCoV-19 vaccination: we found an average age of 44 years, demonstrating a high rate of involvement of females under 50 y.o.

In 8/10 cases of BNT162b2 administration, through the data collected during autopsy, the authors [29,30] excluded the causality relationship considering the previous comorbidities of all involved subjects. Two cases are of interest for the scientific community: two deceased subjects tested positive for the COVID-19 infection. Although in both cases no signs of COVID-19 complications were found, these cases could be related to the SARS-CoV-2 variants that may allow the virus to escape host immunity, in particular, the immunity conferred by vaccination [44,45]. A direct relationship between vaccination and fatal adverse effects was reported by Choi et al. [31] who identified myocarditis as a cause of death: these findings were confirmed in a recent report describing an increased risk of myocarditis in subjects vaccinated with BNT162b2 [46].

Considering the data about the other mRNA vaccine (mRNA-1273), Verma et al. [32] reported the first fatal case after the second rather than first dose, although the same authors reported that a direct causal relationship cannot be definitively established because they did not perform testing for viral genomes or auto-antibodies in the tissue specimens.

Finally, the two cases related to the Ad26.COV2.S (Janssen) vaccine were classified as a “possible” relationship with the COVID-19 vaccination. Considering that the Ad26.COV2.S (Janssen) vaccine is based on a specific type of adenovirus, it is important to note that the anti-PF4 heparin antibody test was positive, similar to the ChAdOx1 nCoV-19 cases.

Although post-mortem investigations were reported in only a few cases, it is reasonable to assume that the potential causality between death and COVID-19 vaccination had been studied in a large number of post-mortem investigations for different reasons that had not been published: the fact that only 17 papers with post-mortem investigations were published does not mean that post-mortem investigations in deaths after vaccination were not performed. This consideration is important in order to clarify the important effort that the scientific community is still making to clarify all aspects related to COVID-19 vaccination.

Vaccines represent some of the greatest medical and scientific achievements of the modern era. In particular, in the pandemic scenario, a medico-legal perspective on vaccination is very important to provide a critical viewpoint. COVID-19 vaccine administration involves different questions, such as the possibility of side effects, which led to a fairly diffuse suspicion and rejection by many people, especially when a fatal case occurring at the same time as vaccine administration occurred in a young healthy subject. The use of vaccines also poses various ethical and legal problems, including the possibility of conflicts between individual and collective rights [34,35].

In a recent report [47], the link between vaccinations and the principles of biomedical ethics has been discussed by assessing four fundamental principles: autonomy (freedom of choice: mandatory and non-mandatory), non-maleficence (not causing harm), beneficence (promoting good), and legality. Several international authors have focused on this topic individually [48,49], suggesting that an international vaccination program should follow seven ethical principles [49]:

- The vaccination plan should concern a disease that represents a public health issue;
- The vaccine should be safe and effective;
- The distress to participants should be as low as possible;
- The benefit/risk ratio of the program must be favorable for participants;
- The immunization program should give the population an equal share of the benefits and burdens;
- The involvement should be, in general, voluntary, except where compulsory vaccination is essential to prevent a real risk;
- Public trust in vaccination programs should be respected and preserved.

In the case of vaccination against COVID-19, different governments decided to vaccinate healthcare personnel as a priority, playing a critical role in infection control in healthcare facilities. Similar decisions have been applied worldwide [50,51,52]. Therefore, considering that most deaths were recorded in the elderly and so-called “fragile” subjects, the vaccine administration was prioritized to these categories, widening the range of subjects involved. In the case of COVID-19 infection, there has been much debate on mandatory vaccination, although at present there is freedom of choice for everyone except for several categories such as health workers. If individual health implies self-determination, i.e., the right of everyone to decide whether and how to treat themselves (in the extreme, even not to treat themselves or let themselves die), in the case of collective health this right may be limited or weakened.

## 5. Key Recommendations

On the basis of the discussed data, we want to suggest several key recommendations to improve awareness of vaccine safety:

- All pathologists should publish autopsy reports in peer-reviewed journals or alternatively, deposit these reports in national/international databases maintained by pathologist societies; in this way, it will be possible to examine the causality relationship worldwide, analyzing other vaccines;
- All pathologists should apply the WHO algorithm to define the causality relationship between vaccination and adverse effects. Analyzing the data of this review, this important tool was usually not applied, although its use is strongly encouraged to define the causality of an adverse event following vaccination (AEFI) [53];
- The scientific community should consider the opportunity to create an international database with all data on adverse effects related to the COVID-19 vaccination that may be implemented and consulted by scientists worldwide.

## 6. Conclusions

In this context, the scientific community must work hard to reduce the growing hesitation to vaccinate among the general population following several cases of fatal adverse reactions. In many cases, the opposition, in the case of vaccines, is linked to pseudo-scientific reasons (not supported by evidence) or utilitarian reasons, therefore, it is not a question of conscientious objection. This raises a very delicate question: to what extent can an adult transfer the possible negative consequences of his or her choices to the entire community, as in the case of COVID-19 vaccination.

The great challenge for the scientific community in the fight against COVID-19 is represented by the success of a global vaccination campaign and, in this light, it is important to provide scientific evidence to remove the doubt of public opinion. In order to avoid another „Lockdown of science” [54,55], we are firmly convinced that autopsy should be the rule in the causality assessment of fatal cases occurring at the same time as COVID-19 vaccination. Measures such as clarifying vaccine safety and effectiveness are essential to reduce vaccine hesitancy in the general population. Despite vaccine hesitancy being a global phenomenon, the causes are very different in each country. As discussed in the present review, the reports about the severe adverse effects of vaccination, such as thrombosis, thrombocytopenia, and myocarditis, have negatively influenced public opinion, slowing down the vaccine program. In line with these considerations, it is desirable that all data collected after post-mortem investigations are shared in the scientific community in order to point out the relative countermeasures.

The development and large-scale implementation of COVID-19 vaccination represents a promising tool to achieve herd immunity, with the possibility to stop this global crisis. Many issues must be addressed regarding current approaches to vaccination to build an effective and correct public health response, building preparedness for future outbreaks.

## Figures and Tables

**Table 1:** The main data of the post-mortem examination performed on cases occurring at the same time as COVID-19 vaccination: on a total of 38 cases, 22 patients were vaccinated with ChAdOx1 nCoV-19, 10 cases with BNT162b2, 4 cases with mRNA-1273, 2 cases with Ad26.COV2.S.

Reference	Vaccine	Fatal Cases				Post-mortem Findings	Causality Relationship	WHO Algorithm
		Sex, Age	D	H	R			
Greinacher et al. [19]	ChAdOx1 nCoV-19	M, 49 y.o.	10	1	n.a.	Cerebral venous thrombosis; portal-vein thrombosis, including the splenic and upper mesenteric veins; in addition, small thrombi were visualized in the infrarenal aorta and both iliac arteries.	YES	NOT DESCRIBED
Althaus et al. [20]	ChAdOx1 nCoV-19	F, 48 y.o.	6	10	n.a.	Complete thrombotic obstruction of the straight, sagittal and transversal cerebral sinuses; subarachnoid hemorrhage; cerebral edema and bilateral pulmonary embolism; obstruction of glomerular arterioles and capillaries by hyaline microthrombi containing fibrin and platelets.	YES	NOT DESCRIBED
		M, 24 y.o.	10	7	Het. FVL	Massive cerebral hemorrhage and cerebral edema, bilateral pulmonary thromboembolism and obstruction of glomeruli by hyaline microthrombi.	YES	NOT DESCRIBED
Mauriello et al. [21]	ChAdOx1 nCoV-19	F, 48 y.o.	18	21	pre-existing condition of thrombocytopenia due to myelodysplasia	Massive cerebral hemorrhage; purulent abscess involving the right fronto-temporo-parietal lobes, the nucleus of the right base, with midline shift and wedging of the cerebellar tonsils and an internal and external hemotocephalus.	YES	NOT DESCRIBED
Wieldmann et al. [22]	ChAdOx1 nCoV-19	F, 34 y.o.	7	1	None	Edematous brain with sparse subarachnoid hemorrhage and a large hemorrhagic infarction in the right hemisphere; thrombi were present in both transverse sinuses.	YES	NOT DESCRIBED
	ChAdOx1 nCoV-19	F, 42 y.o.	10	15	n.a.	Thrombus in the left transverse and sigmoid sinus, as well as in the sagittal cerebral sinus; massive hemorrhagic infarction in the left hemisphere; peripheral areas with infarction in the lungs.	YES	NOT APPLIED
	ChAdOx1 nCoV-19	F, 37 y.o.	8	3	n.a.	Large hemorrhagic infarction in the left cerebral hemisphere; extensive hemorrhagic changes in the cerebellum, as well as focal white substance hemorrhages in the cerebral hemispheres and in the brainstem. Thrombi were present in the left transverse and sigmoid sinuses.	YES	NOT DESCRIBED
	ChAdOx1 nCoV-19	F, 54 y.o.	6	2	n.a.	Thrombi in the posterior sagittal sinus and both transverse sinuses. Massive hemorrhagic venous infarction in the right parietal lobe and bilateral hemorrhagic infarctions in multiple cortical areas.	YES	NOT DESCRIBED
Bjørnstad-Tuveng et al. [23]	ChAdOx1 nCoV-19	F, n.a. (young)	7	n.a.	None	Intracranial hemorrhage. Moreover, small thrombi were found in the transverse sinus, frontal lobe, and pulmonary artery.	YES	NOT DESCRIBED
Scully et al. [24]	ChAdOx1 nCoV-19	F, 55 y.o.	6	n.a.	n.a.	Thrombosis in many small vessels, especially vessels in the lungs and intestine, cerebral veins, and venous sinuses, as well as evidence of extensive intracerebral hemorrhage.	YES	NOT DESCRIBED
Günther et al. [25]	ChAdOx1 nCoV-19	M, 54 y.o.	12	1	None	Residual thrombus in the left sinus transversus; no evidence for other thromboembolic pathology in the brain or other solid organs was found.	YES	NOT DESCRIBED
Pomara et al. [26,27]	ChAdOx1 nCoV-19	M, 50 y.o.	10	6	None	Portal and mesenteric thrombosis with extension into the splenic vein. Moreover, extensive cerebral hemorrhages were described.	YES	YES
		F, 37 y.o.	13	10	None	Thrombi in cerebral sinus; massive thrombosis of the whole venous tree of left upper limb extending from the hand to the axillary vein, with symmetric lesions in the veins of the right hand and the right axillary vein.	YES	YES
Schneider et al. [28]	ChAdOx1 nCoV-19	F, 32 y.o.	12	Home	None	Massive cerebral hemorrhage, anti-PF4 heparin antibody tests: positive, HIPA-Test: positive, PIPA-Test: positive.	Very likely	NOT DESCRIBED
	ChAdOx1 nCoV-19	F, 34 y.o.	1	Home	Obesity, massive cardiac hypertrophy, myocardial infarction scars	Recurrent myocardial infarction in the presence of massive cardiac hypertrophy.	NO	NOT DESCRIBED
	ChAdOx1 nCoV-19	F, 48 y.o.	10	Workplace	None	Aortic dissection with rupture, high blood loss.	NO	NOT DESCRIBED
	ChAdOx1 nCoV-19	M, 63 y.o.	14	Home	Severe pre-existing cardiac changes	Severe coronary sclerosis, cardiac hypertrophy, myocardial infarction scars, liver cirrhosis.	NO	NOT DESCRIBED
	ChAdOx1 nCoV-19	M, 61 y.o.	1	Home	Severe pre-existing cardiac changes	Severe coronary sclerosis, massive cardiac hypertrophy, negative anaphylaxis diagnostics.	NO	NOT DESCRIBED
	ChAdOx1 nCoV-19	M, 71 y.o.	10	Home	Severe coronary sclerosis, massive cardiac hypertrophy, myocardial infarction scars	Pulmonary embolism in the presence of Deep Vein Thrombosis.	NO	NOT DESCRIBED
	ChAdOx1 nCoV-19 (second dose)	F, 38 y.o.	8	Hospital (n.a.)	n.a.	Multiple fresh thrombi, including in the cerebral venous sinuses, cardiac hypertrophy, fresh myocardial infarction, hypoxic brain damage, anti-PF4 heparin antibody tests: positive, HIPA-Test: positive, PIPA-Test: positive.	Unlikely	NOT DESCRIBED
	ChAdOx1 nCoV-19	F, 65 y.o.	10	Hospital (n.a.)	n.a.	Signs of a bleeding diathesis, cerebral hemorrhages, CVT, mild coronary sclerosis, anti-PF4 heparin antibody tests: positive, HIPA-Test: positive, PIPA-Test: positive.	Very likely	NOT DESCRIBED
	ChAdOx1 nCoV-19	M, 57 y.o.	2	Hospital (n.a.)	Massive cardiac hypertrophy	Severe coronary sclerosis, extensive myocardial infarction scars, fresh myocardial infarction.	NO	NOT DESCRIBED
Edler et al. [29]	BNT162b2	F, n.a. (elderly)	5	0	Coronary heart disease, cardiac insufficiency, arterial hypertension, dementia and hyperthyroidism.	Pulmonary artery embolism with infarction of the right lower lobe of the lung with deep leg vein thromboses on both sides.	NO	NOT DESCRIBED
			She was found dead					
		M, n.a. (elderly)	10	2	Chronic renal failure, anemia, atrial fibrillation, pulmonary artery embolism, arterial hypertension, peripheral artery disease, right thalamic infarction with left hemiparesis, recurrent tonic-clonic seizures, gait disorder with polyneuropathy, rheumatoid arthritis and prostate carcinoma with prostatectomy.	Nasopharyngeal swab for SARS-CoV-2 RNA was positive. Autopsy revealed chronic and acute pancreatitis. Pneumonia was confirmed as the cause of death.	NO	NOT DESCRIBED
		M, n.a.	2 (he was found dead)	0	Apoplexy and myocardial infarction as well as arterial hypertension and type II diabetes mellitus.	The known pre-existing conditions were confirmed, and further organ pathologies typical of old age were found in the form of signs of chronic obstructive pulmonary disease (COPD) and chronic renal dysfunction.	NO	NOT DESCRIBED
Hansen et al. [30]	BNT162b2	M, 86 y.o.	18	7	Past medical history included systemic arterial hypertension, chronic venous insufficiency, dementia and prostate carcinoma.	Nasopharyngeal swab for SARS-CoV-2 RNA was positive (day 24). No characteristic morphological features of COVID-19 were reported (i.e., alveolar damage in the lungs); extensive acute bronchopneumonia, possibly of bacterial origin.	NO	NOT DESCRIBED
Schneider et al. [28]	BNT162b2	M, 65 y.o.	1	Home	Severe pre-existing cardiac changes	Severe coronary sclerosis, massive cardiac hypertrophy, myocardial infarction scars, myocarditis.	POSSIBLE	NOT DESCRIBED
	BNT162b2	M, 71 y.o.	1	Home	Severe pre-existing cardiac changes	Massive cardiac hypertrophy, coronary sclerosis, negative anaphylaxis diagnostics.	NO	NOT DESCRIBED
	BNT162b2	F, 72 y.o.	12	Home	Coronary sclerosis, cardiac hypertrophy	Massive cerebral hemorrhage.	NO	NOT DESCRIBED
	BNT162b2 (second dose)	M, 79 y.o.	6	Home	Deep Vein Thrombosis	Massive pulmonary embolism, coronary sclerosis, pericarditis, chronic pulmonary Emphysema.	NO	NOT DESCRIBED
	BNT162b2 (second dose)	F, 72 y.o.	0	Vaccination center	n.a.	Severe coronary sclerosis with coronary thrombosis, myocardial infarction scars, fresh myocardial infarction.	NO	NOT DESCRIBED
Choi et al. [31]	BNT162b2	M, 22 y.o.	5	7 h	None	On microscopic examination, diffuse inflammatory infiltration, with neutrophil and histiocyte predominance was observed within the myocardium. Notably, the inflammatory infiltrates were dominant in the atria, and around the sinoatrial (SA) and atrioventricular (AV) nodes, whereas the ventricular area displayed minimal or no inflammatory cells. Occasional myocyte necrosis or degeneration was found adjacent to the inflammatory infiltrates, without abscess formation or bacterial colonization. The cause of death was determined to be myocarditis.	YES	NOT DESCRIBED
Verma et al. [32]	mRNA-1273 (second dose)	M, 42 y.o.	15	7	None	An inflammatory infiltrate admixed with macrophages, T-cells, eosinophils, and B cells was observed in heart tissue. The cause of death was defined as fulminant myocarditis that had developed within 2 weeks after COVID-19 vaccination.	YES	NOT DESCRIBED
Schneider et al. [28]	mRNA-1273	M, 82 y.o.	1	Home	Pre-existing cardiac changes with infarction	Severe coronary sclerosis, massive cardiac hypertrophy, extensive myocardial infarction scars, negative anaphylaxis diagnostics.	NO	NOT DESCRIBED
	mRNA-1273	F, 91 y.o.	1	Home	Pre-existing cardiac changes with infarction	Severe coronary sclerosis, massive cardiac hypertrophy, myocardial infarction scars, negative anaphylaxis diagnostics.	NO	NOT DESCRIBED
	mRNA-1273 (second dose)	F, 57 y.o.	6.	Home.	Hyperglycemic coma	Severe coronary sclerosis, fatty liver, high levels of glucose and lactate in cerebrospinal fluid (CSF) and aqueous humor exceeding the cumulative levels of Traub.	NO	NOT DESCRIBED
Schneider et al. [28]	Ad26.COV2.S (Janssen)	M, 69 y.o.	9	Home	n.a.	CVT, severe coronary sclerosis with coronary thrombosis, massive cardiac hypertrophy, fresh myocardial infarction, anti-PF4 heparin antibody tests: positive, HIPA-Test: positive, PIPA-Test: positive.	POSSIBLE	NOT DESCRIBED
Choi et al. [33]	Ad26.COV2.S (Janssen)	M, 38 y.o.	2	10 h	Smoldering multiple myeloma had been diagnosed 1.5 years before	Autopsy results showed no evidence of acute infection or cardiovascular disease in the internal organs. Moreover, pulmonary edema, pleural effusion, and pericardial effusion were reported.	POSSIBLE	NOT DESCRIBED

Legend: (D) first symptoms after vaccination (days); (H) Hospitalization (days); (R) clinical features; (n.a.) not available/performed; (FVL) Factor V Leiden; (Het.) Heterozygous.

## Data Availability

All data generated or analyzed during this study are included in this published article.
